# Bacteriology

**Published:** 1899-11

**Authors:** 


### Bacteriology.

Bacteriology in its application to diagnosis and treatment in practical medicine is yet in its infancy; but it is a very robust infancy, full of promise, the complete fulfilement of which none of use will live to see. The bacterial origin of tubercle, anthrax, diphtheria, erysipelas, septicemia, typhoid, malaria and influenza, has been revealed to us almost within the memory of the youngest, yet has already been, in many instances, fruitful in suggesting measures of prevention and treatment. Bacteriology, in all its departments, is and must ever remain subject to expert investigation. It is impossible for the busy practitioner to find the time or to maintain the technical skill and apparatus necessary for trustworthy investigation. The various research associations have hitherto in part fulfilled the want and the bacteriologic departments of our hospitals are steadily growing in importance and value; but it is to be hoped that the time will soon come when in every district throughout the country there will be in connection with the public health department a bacteriologic laboratory, where the ordinary, and even the extraordinary, clinical tests will be at the command of every practitioner at a moderate scale of fees.

